# Surgical resection of thalamic tumors: a report of four cases

**Published:** 2012-11

**Authors:** Hassan Reza Mohammadi, Parisa Azimi

**Affiliations:** ^a^Department of Neurosurgery, Shahid Beheshti University of Medical Sciences, Tehran, Iran.

## Abstract

Thalamic tumors (TTs) are rare which represent about 4% of all brain tumors.

**Case:**

The present study reports four cases of TTs Of which 3 cases were male and 1 female, aged 10 to 20 years (mean age= 14.2 years). Symptoms of raised intracranial pressure (75%), and hemiparesis (50%) were the most frequent manifestations in our TTs patients. All patients had a low-grade glioma. All cases were operated through transcortical or transsylvian-transinsular approaches. Gross-total resection was accomplished in 3 patients and subtotal resection (STR) in 1. Although hydrocephalus was frequent, only 2 patients required a ventriculoperitoneal shunt placement. After the follow-up period (average time of 12 months, range 3–24 months), adjuvant therapy was not administered for our patients. At the last clinical visit, 4 patients were alive, of which 3 cases showed the normal neurological examination results. The outcomes for the remaining patients were classified as mild (n=3) and moderate disability (n=1).

**Keywords:**

Brain tumors, Thalamic Tumors, Surgery


					Volume 4, Suppl. 1 Nov 2012
Publisher: Kermanshah University of Medical Sciences
URL: http://www.jivresearch.org
Copyright:
Congress of Iranian Neurosurgeons, 14-16 November 2012, Kermanshah, Iran
Paper No. 5
Surgical resection of thalamic tumors: a report of four cases
Hassan Reza Mohammadi a
, Parisa Azimi a,*
a
Department of Neurosurgery, Shahid Beheshti University of Medical Sciences, Tehran, Iran.
Abstract:
Thalamic tumors (TTs) are rare which represent about 4% of all brain tumors.
Case: The present study reports four cases of TTs Of which 3 cases were male and 1 female, aged 10 to 20 years
(mean age= 14.2 years). Symptoms of raised intracranial pressure (75%), and hemiparesis (50%) were the most
frequent manifestations in our TTs patients.. All patients had a low-grade glioma. All cases were operated through
transcortical or transsylvian-transinsular approaches. Gross-total resection was accomplished in 3 patients and
subtotal resection (STR) in 1. Although hydrocephalus was frequent, only 2 patients required a ventriculoperitoneal
shunt placement. After the follow-up period (average time of 12 months, range 3-24 months), adjuvant therapy was
not administered for our patients. At the last clinical visit, 4 patients were alive, of which 3 cases showed the normal
neurological examination results. The outcomes for the remaining patients were classified as mild (n=3) and
moderate disability (n=1).
Keywords:
Brain tumors, Thalamic Tumors, Surgery
* Corresponding Author at:
Parisa Azimi: Department of Neurosurgery, Imam-Hossain Hospital, Shahid-Beheshti University of Medical Sciences, Imam-Hossain sq., Tehran, Iran.
Tel: 021-77558081, Email: Parisa.azimi@gmail.com, (Azimi P.).

				

